# Managing Distress Using Mobile Prescriptions of Psychological Pills: A First 6-Month Effectiveness Study of the PsyPills App

**DOI:** 10.3389/fpsyt.2019.00201

**Published:** 2019-05-01

**Authors:** Oana A. David, Daniel David

**Affiliations:** ^1^Department of Clinical Psychology and Psychotherapy, The International Institute for the Advanced Studies of Psychotherapy and Applied Mental Health, Babes-Bolyai University, Cluj-Napoca, Romania; ^2^Department of Oncological Sciences, Mount Sinai School of Medicine, New York, NY, United States

**Keywords:** stress management, mobile mental health, computerized CBT, psychological pills, emotion-regulation, cognitive change strategies, functional reappraisal

## Abstract

**Background:** Although numerous mental health apps are commercially available, only a few of them have been empirically tested. PsyPills is an interactive and personalized mobile application, based on emotion regulation research and Rational Emotive Behavior Therapy principles, that can function as a stand-alone intervention aimed at offering immediate stress relief.

**Objective:** In this paper, we describe the newly developed PsyPills app and present data obtained at 6 months after its release regarding its effectiveness for stress management.

**Methods:** 115 users aged 15–79 years old (*M* = 39.01, *SD* = 13.49) accessed the app during the first 6 months after its release and were thus included in the study. Distress and specific cognitive processes were collected using visual analog scale measures.

**Results:** Most users accessed the app with the purpose of searching anxiety relief and most often reported work-related distress. Seventy-Four users accessed PsyPills between 1 and 11 times (*M* = 2.68, *SD* = 2.59), and received 258 psychological prescriptions in total. PsyPills was effective in terms of reducing the frequency of dysfunctional emotions, such that significantly more users reported feeling functional emotions after accessing the application and reading its personalized prescriptions than those reporting not being able to change it [χ^2^ (1, *N* = 52) = 52.00 *p* < 0.001]. Using reminders of the psychological pill at specific times during the day made the PsyPills app more effective.

**Conclusions:** Based on initial data on its first 6-month usage, the PsyPills app appears to be promising in terms of offering stress relief. However, future studies need to use golden standard design and investigate its efficacy as an adjunctive intervention.

## Introduction

Emotional disorders have been conceptualized as “distress disorders” (1) and are widely viewed as the result of difficulties in regulating emotional distress (2). Individuals who cannot effectively regulate their emotional responses to everyday events experience longer and more severe periods of distress that may evolve into diagnosable mental illness [e.g., (2, 3)]. Since mental illness has a huge negative impact on the individual and societal functioning of the person, it becomes imperious to find new ways to improve access to evidence-based interventions aimed at promoting mental health and helping people cope when in distress.

Cognitive-behavioral therapy (CBT) has proven to be effective in the treatment and prevention of emotional disorders [see Driessen and Hollon (3); see also NICE guidelines (4)]. Therefore, efforts have been made to increase accessibility to CBT based interventions for those in need of managing stress and as a means to prevent emotional disorders. Internet and computer-based cognitive-behavioral therapy (i/cCBT) intervention protocols are widely available and have been found to yield comparable results to face-to-face CBT, both in adult and youth samples (5).

In particular, mobile-based mental health applications are adding increased benefits compared to face to face treatment, such as higher accessibility (e.g., ecological assessment), while offering comparable functionalities to those of computers. The number of mental health mobile applications has grown exponentially in the past years (6). However, few of them have been tested empirically. A review by Donker et al. (7) found eight studies describing the effectiveness of five mobile applications in the treatment of depression, anxiety, and substance abuse. The authors found a high variability in terms of the benefits reported in the studies, with effect sizes ranging from small to large. A qualitative review conducted by Torous and Powell (8) presents 10 studies documenting the effectiveness of mobile-based interventions in managing depressive disorders and suggests that more research is needed in order to draw firm conclusions. Benefits have been also noted for the use of mobile mental health apps for stress monitoring (9), with most stress monitoring apps being designed for workplace settings. A recent review by Menon et al. (10) included eight studies and found support for the effectiveness of psychotherapeutic mobile based interventions on the main mental health outcomes. The authors suggest that future studies investigate their effectiveness in real world settings, especially in low and middle-income countries.

## Objectives and Hypotheses

The focus of this paper is on presenting initial data regarding the effectiveness of the newly developed PsyPills mobile application in improving stress management in users. PsyPills is the first mental health mobile application integrating the principles of Rational Emotive Behavior Therapy (REBT; 11) in an interactive and personalized format. PsyPills is grounded in positive psychology and emotion regulation research and is meant to be used as a standalone intervention for stress management. We hypothesized that its users would report lower distress after using the application, compared to initial levels. We also expected that users accessing its help resources (namely psychoeducation and reminders) would obtain better results by post-test.

## Methods

### Design

Since CBT/REBT interventions are already supported by RCSs, we considered that an effectiveness trial will offer us important data regarding the external validity and user acceptance of the application. Thus, we used a one group pretest-post-test comparison design to investigate the real-world effectiveness of the PsyPills app 6 months after its release.

### The Psypills App

**Figure 1:**
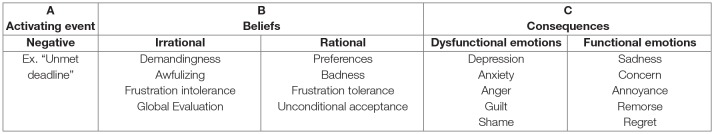
Sequence of the screens in the PsyPills app.

The PsyPills app is based on the concept of “Psychological pills”, an innovative concept introduced by David (11). The analogy with the medical field implied by the name is meant to underline the application's evidence-based approach, drawing a parallel to personalized medicine. The application offers personalized prescriptions of emotion regulation strategies for coping with distress, based on evidence-based CBT and positive psychology protocols. Rational Emotive Behavior Therapy (REBT) is a specific CBT approach developed by Ellis (12), which focuses on restructuring faulty evaluative cognitions (irrational beliefs) and strengthening rational beliefs in order to change dysfunctional emotions (distress) and maladaptive behaviors [see David et al. (13)]. The REBT approach is based on the ABC model [(14–16); see Figure 1], which can be summarized as follows: when individuals are faced with negative activating events (A), they have certain beliefs (B) about these events, which then mediate the emotional or behavioral consequences (C) of these events. As such, when their beliefs (B) are rational/functional, their emotional and behavioral consequences (C) will be adaptive (or functional); in turn, if their beliefs (B) are irrational/dysfunctional, their emotional and behavioral consequences (C) will be maladaptive (or dysfunctional) [see Visla et al. (17)].

REBT theory was chosen for the application since is consistent with findings from the emotion regulation paradigm indicating that reappraisal strategies—changing how one evaluates events in order to change emotional responses (18)—are effective in the treatment of psychopathology [see Aldao et al. (19)] and in dealing with distress (20, 21). Moreover, similarly to the Beckian approach, Ellis's positive theory of cognitive mechanisms of mental health refers to rational counterparts to irrational processes (see Figure 1) prescriptive format of the PsyPills.

The first module uses standardized assessment to evaluate the level of distress experienced by the user during the previous week and ecological assessment procedures (also available as a free teaser, the MoodWheel app) to record the user's current mood. In the PsyPills app, distress is conceptualized based on the binary model of distress (22, 23) in terms of dysfunctional negative emotions (such as depressed mood, anxiety, anger) corresponding to maladaptive behaviors and dysfunctional/irrational thinking (see Figure 1).

The aim of the app is to teach the users how to change their dysfunctional negative emotions into functional negative emotions, by using rational thinking strategies, which, although negative, are adaptive for their negative situation and prepare them for finding other resources based on positive psychology thinking strategies (optimism). Although, based on the binary model of distress, reducing the intensity of a negative emotion can offer stress relief, it is more important to obtain a qualitative change in terms of transforming a dysfunctional emotion into a functional one, so as to maintain motivation for change when in a negative situation.

Depending on the specific dysfunctional negative emotion that the user is reporting, he/she receives a personalized rational statement (called functional reappraisal in the emotion-regulation paradigm) to internalize, in order to change his/her mood into a functional emotion. The prescription also includes recommendations regarding the administration rate of the “psychological pill,” depending on the user's needs. The statement format included in the PsyPills was previously tested in rigorous studies, based on the emotion regulation paradigm (20, 21, 24–26). Using the application, help can be accessed in a rapid and interactive way: the user is required to select from specific options regarding cognitive processes involved in the emotion experienced, and based on three quick selections, the app offers a personalized “psychological pill” within a prescription. The user is requested to read the prescriptions at specific times/when they feel dysfunctional negative emotions. The app also gives the user access to psychoeducational information regarding the relationship between thinking patterns and stress.

### Participants

One hundred and Fifteen users aged 15–79 years old (*M* = 39.01, *SD* = 13.49) accessed the app during the first 6 months after its release and thus were included in the study. We used Facebook posts and a page dedicated to the app, media presentations (newspapers, TV shows) and professionals group posts as recruitment strategies. 57.39% of the users were males and 56.37% were married. The majority (88.87%) were White Caucasians, the rest being African American, Hispanic, and Asian. 25.22% accesed the app from the USA, 26.09% accessed it from Romania, while the rest were spread around the globe with low percents. In terms of the education levels, 11.3% had high school level studies, 20.87% had a Bachelor degree, 33.91% had a Master degree, 14.78% a doctorate, and 19.13% post-doctoral studies. Most of the participants were psychologists (35.62%), followed by psychotherapists (16.74%), medical doctors and teachers (6.85% each), social workers (5.48%) economists, engineers, coaches (4.11% each), human resource employees (2.74% each), students (7.85%), researchers, and aviators (less than 1.5%).

### Procedure

**Figure 2:**
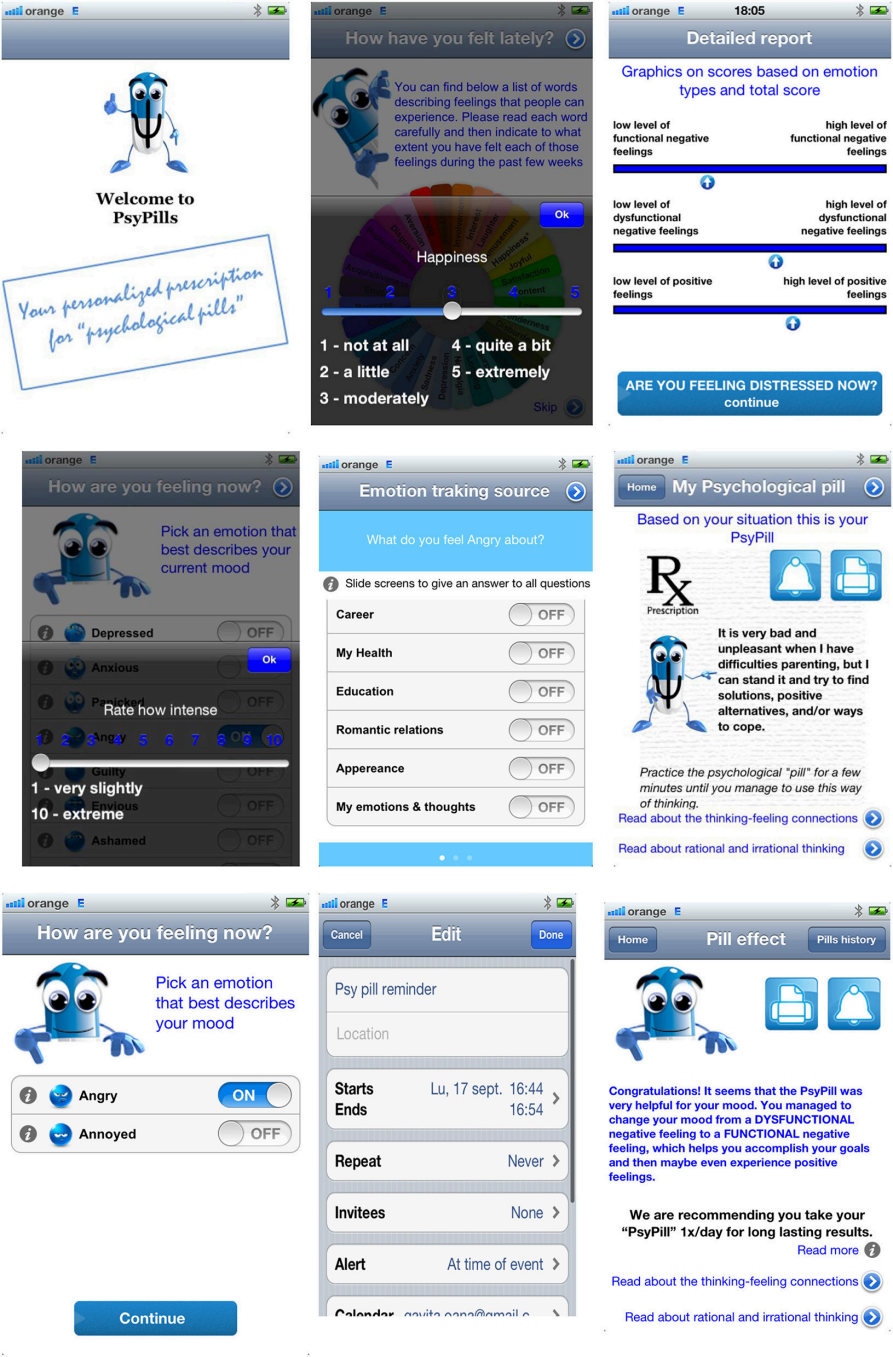
The ABC model of REBT and the main irrational and rational processes. Sequence of the screens in the PsyPills app.

The study was approved by the Ethical Committee of the authors' affiliate University. Participants filled the consent form as part of the sign-up process and agreed for their input data to be used in an anonymous way for research purposes. The app uses a platform that stores all user data. Upon first time access, users filled in a mood measure, based on which the app displayed a graph of their mood during the past few weeks. The app recorded users' current mood as pre-assessment. Participants completed the pre-test assessment each time they accessed the app and were required by the app to complete the post-test assessment in the same session, after using the prescription. The app used the algorithm described above to generate a personalized psychological pill each time. For example, when a participant selected feeling distressed over the area of parenting, the cognitive content of comfort, the process low frustration tolerance, the PsyPills would generate the following psychological pill: “It is very unpleasant when I have difficulties with parenting, but I can stand it and try to find solutions, positive alternatives, and/or ways to cope.” Participants were able to set reminders to read their psychological pill or to print it (see Figure 2).

### Measures

The main outcome of the study are negative emotions (dysfunctional and functional). Assessment of the cognitive processes was only carried out initially for descriptive analyses.

#### Emotions

Visual Analog Scales were used for rating users' current dysfunctional and functional emotions. Initially, users were asked to respond to the question: “How do you feel now?” and instructed to pick the emotion that best described their current mood from a list of nine dysfunctional emotions (depressed mood, anxiety, panic, shame, anger, guilt, jealousy, envy, hopeless). Then they were asked to rate the intensity of the emotion on a scale from 1 to 10, where 1 meant very slightly and 10 referred to an extremely intense emotion. After receiving the personalized prescription, the user received the same instructions, with the exception that they had to choose between the specific dysfunctional negative emotion initially selected vs. its functional alternative (e.g., depressed or sad; anxious or concerned), and rate its intensity again.

#### Cognitive Processes

Cognitive processes are assessed in the section Emotion tracking source, with a view to extracting the personalized “psychological pill” and include it in the prescription given to the user. The three questions used for this purpose refer to: (1) The domain of the emotion (What do you feel [selected emotion] about?) with the option of selecting from 12 life domain areas; (2). Irrational processes, where the user can select from six well established irrational processes in the literature [see David (27)]: Demandingness, Awfulness, Frustration Intolerance, Global Evaluation of Self, Global Evaluation of Other, and Global Evaluation of Life; (28). Belief content, with four options representing well documented relevant content areas in the literature [see David (27)]: achievement, approval, comfort, and fairness.

## Data Analysis

For the inferential analyses, we used the Chi square test, to compare the differences in the proportion of participants reporting functional vs. dysfunctional emotions at post-test. We also compared the intensity of dysfunctional emotions reported after using the app with initial levels using a paired *t-*test.

## Results

### Descriptive Statistics

Seventy-Four of the users accessed pills between 1 and 11 times (*M* = 2.68, *SD* = 2.59). Two Hundred and Fifty-Eight pills were prescribed and users re-rated emotions after receiving their personalized “psychological pill” 101 times. Fifty-Four of the users re-rated their emotion after receiving the prescription and we considered their last rating as post-test assessment (see Table 1). Distribution of usage by country is presented in Table 2.

**Table 1:** Descriptive statistics on the intensity of the dysfunctional emotion rated before and after the prescription.

**Variables**	***N***	**Min**	**Max**	***M***	***SD***
Dysfunctional negative emotions pre-test	74	1	10	5.08	2.55
Dysfunctional negative emotions post-test	20	1	10	5.38	2.51
Functional negative emotions post-test	32	1	9	4.34	2.33

*Intensity of the dysfunctional emotions rated before and after the pill – p > 0.05*.

**Table 2:** Countries from where participants accessed the PsyPills app.

**Country**	**Percentage of users (%)**
Aland Islands	0.8
Albania	12.17
Algeria	5.2
Antarctica	0.87
Bangladesh	0.87
Belgium	0.87
Bolivia	0.87
Brazil	1.74
Cambodia	0.87
Canada	4.35
Germany	0.87
Israel	1.74
Japan	0.87
Lebanon	0.87
Norway	0.87
Portugal	0.87
Romania	25.22
Spain	0.87
Switzerland	1.74
Turkey	1.74
United Kingdom	9.57
United States	26.09

### Emotions and Cognitive Processes

In terms of the most frequently reported dysfunctional emotion for which the app was used, anxiety was the most frequent (31.78% of the users), followed by depressed mood (22.48%), anger (16.28%), guilt (7.75%), panic (7.36%), jealousy (5.43%), shame (4.65%), and last envy and hopelessness.

**Figure 3:**
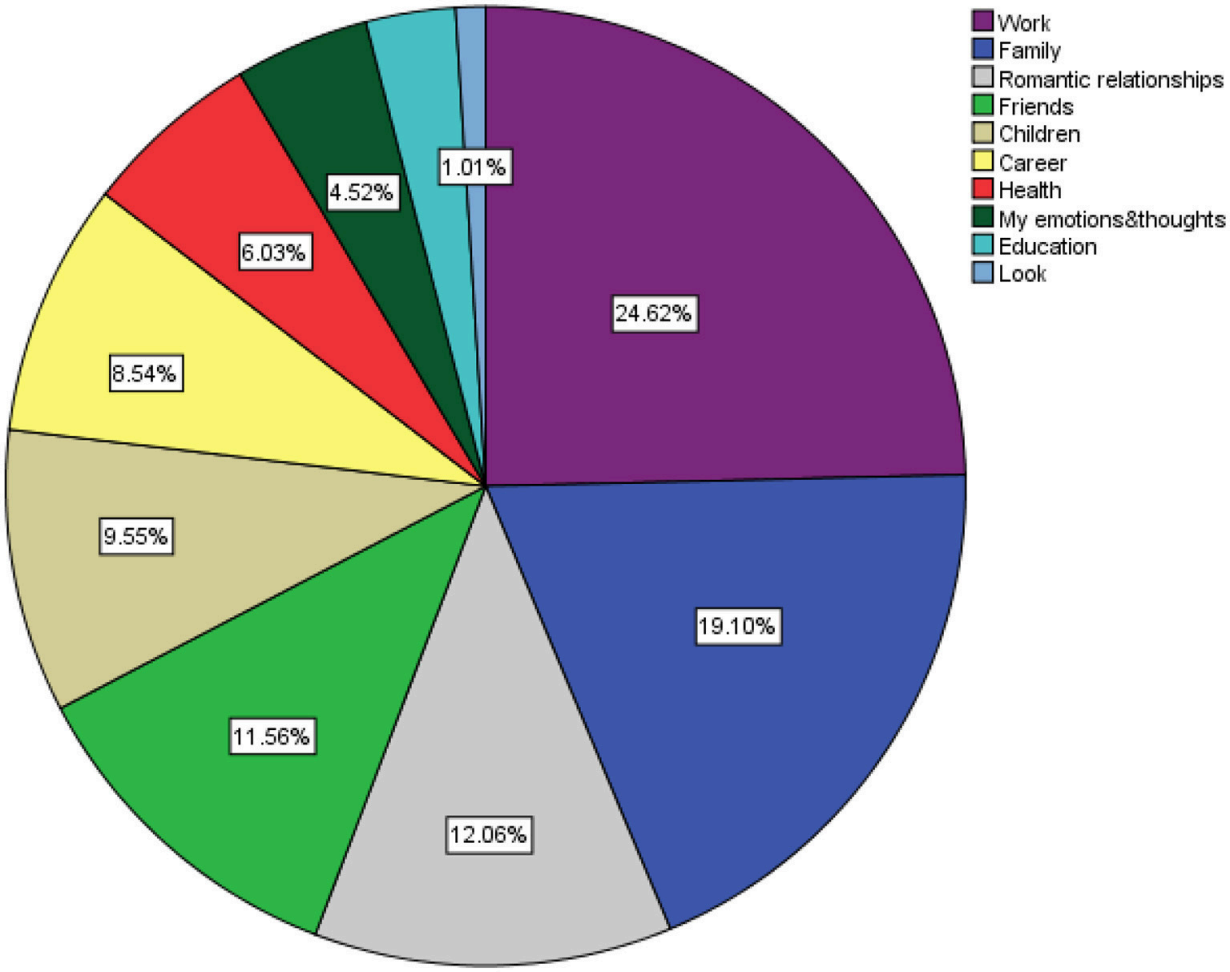
Life domains users reported feeling distress about.

In terms of the life domains users felt distress about, work- (24.62%) and family- (19.10%) related issues were most frequently reported, followed by romantic relationships, friends, children, career, health, mental states, education, and looks (see Figure 3).

In terms of the frequency of the irrational cognitive processes reported by the users, most of the users reported Awfulizing (28.14%) and Global Evaluation (28.14%; 16.08% self-downing), followed closely by Demandingness (25.63%) and Frustration intolerance (18.09%). In terms of the content areas of irrational cognitions, achievement was most frequently reported (32.16%), followed by comfort (28.64%), approval/rejection (24.12%), and fairness (15.06%).

### Psycho-Education

44.35% of the users accessed additional educational information about the nature of functional/dysfunctional feeling, when rating their mood, accessing the screen at least once (24.35%) up to eight times. 30.43% of the users accessed additional information about the Thinking-Feeling connection at least once (20%) and up to five times. 23.48% of the users accessed the screen with information about rational and irrational thinking at least once (12.17%) up to six times.

### Inferential Analyses

#### Dysfunctional vs. Functional Negative Emotions at Post-test

61.54% of the users who re-rated their emotions after using the “psychological pill” reported feeling a functional negative emotion and not a dysfunctional one (as they had initially). We obtained a significant difference in terms of the frequency with which app users reported feeling a functional negative emotion compared to the frequency with which users still reported feeling a dysfunctional negative emotion at post-test [χ^2^(1, *N* = 52) = 52.00 *p* < 0.001)].

We did not obtain any significant differences in terms of the intensity of dysfunctional emotions reported after using the app (post-test) compared to the initial levels of dysfunctional emotions (*p* < 0.05).

### *Post-hoc* Moderation Analyses

Based on the few studies in the field we were not able to make specific predictions regarding the characteristics of the app or population that would make it more effective and thus we tested the potential moderating effects of those variable as *post-hoc* analyses. We tested a potential moderation effect played by the age of the users (with younger users potentially having better results) and the use of app, such as times used, usage of psycho-education information or reminders, in terms of the effectiveness of the PsyPills. We expected that repeatedly using the app and making use of the resources offered by the app would improve users' results, due to a more in-depth understanding and practice of adaptive thinking strategies.

We did not find a moderation effect in terms of the number of times users accessed the app [*F*_(7, 31)_ = 0.99, *p* = 0.45], users' age or the access to psychoeducation sections (all *p*s > 0.05). We found a marginally significant difference in terms of lower levels of dysfunctional negative emotions [*F*_(1, 44)_ = 3.73, *p* = 0.059], and a significantly lower level of functional negative emotions [*F*_(1, 30)_ = 5.51, *p* = 0.026] reported at post-test by those who used the reminder option of the app.

## Discussions and Conclusion

We tested the effectiveness of a newly developed stress management app, PsyPills, during the first 6 months after its release. The app is grounded on the REBT theory and research and is in line with findings from the emotion-regulation paradigm, emphasizing the efficacy of functional reappraisal strategies for mood management (20). Our results show that most users accessed the app with the purpose of searching anxiety relief and most often reported work-related distress. In terms of the cognitive processes reported in relation with their distress, users most often identified awfulizing as an irrational cognition, and achievement as a type of cognitive content. These findings are similar with recent findings on the connection between specific types of irrational beliefs and dysfunctional emotions [see Visla et al. (17)], and on the ABC model (27), which showed that when working on personal development, anxiety is the second most frequent reported emotion and work-related events are the most frequent.

We expected that the app would help users transform their dysfunctional negative emotions into more functional ones, which could help them cope with the negative events they faced. In line with our expectations, we have found that the PsyPills app was effective in terms of reducing the frequency of dysfunctional emotions, such that significantly more users reported feeling functional emotions after accessing it and reading its personalized prescription than those reporting not being able to change them. In other words, significantly more functional then dysfunctional emotions were reported by participants after using PsyPills. However, the users who were not able to change their emotions into more functional ones did not report a lower intensity of their dysfunctional emotions, compared to their initial level. This finding is in line with the binary model of distress (14) and with our hypotheses but could also suggest the fact that there are certain features that are helpful only to some users. Based on the binary model of distress, functional emotions are qualitatively distinctive from the dysfunctional ones and not quantitatively, like the unitary model of distress suggests. Thus, our results support the assumption that in order to be effective in managing their stress, users needed to change their dysfunctional emotions into functional ones. Also, another possibility is that there might be dysfunctional emotions that are more resistant to change than others; future controlled studies could investigate this hypothesis.

In terms of moderator effects, we expected that specific features of the app, such as repeated usage, psychoeducation or reminders would make it more effective. We only found support for using reminders of psychological pills at specific times during the day. In other words, participants using reminders reported lower intensities of both functional and dysfunctional emotions after using the app. However, it is not clear if those users who were able to manage their mood were more likely to set reminders or if setting reminders was helpful *per se*. Future studies will need to specifically test these possibilities. Our results need to be interpreted cautiously, however, due to some important methodological limitations, such as the real-life design which implies the absence of a control group, and the fact that this study makes use of no validated outcome measures. Also, a considerable proportion of the users were psychologists/psychotherapists and the app might be particularly helpful for them since it could build on their existing skills; thus, future studies need to rely more on the general population, in order to be able to generalize results. Also, since the primary stress-inducing trigger was work, further piloting should focus on its effects in other contexts. Furthermore, we used VAS type of measures for outcomes and future studies could benefit from using standardized measures of psychological stress.

In sum, results of the PsyPills usage during the first 6 months are promising in terms of its effectiveness in building stress management skills. However, for drawing stronger conclusions regarding its efficacy, future studies need to use a gold standard design, such as a randomized clinical trial, and account for variables such as time of user access.

An important limitation of the currently investigated apps for mental health is their availability for users. Donker et al. (7) found that out of the five apps investigated in the literature, only two were available for purchase. The important benefit offered by the PsyPills app is that the app is easy to access and use, and it offers low cost access to evidence-based strategies for stress management. Given the fact that the app also provides access to features such as psychoeducation and ecological momentary assessment, future studies could investigate its efficacy as an adjunctive to psychotherapy.

To conclude, we have found that the PsyPills app is effective in providing immediate stress relief in a variety of life domains. While there are a variety of stress management apps currently available, we do not know if they are using sound theories and how effective they are. The PsyPills app helps to overcome these gaps and gives access to innovative personalized evidence-based stress management strategies.

## Ethics Statement

This study was carried out in accordance with the recommendations of the Ethical Committee of the Babes-Bolyai University with written informed consent from all subjects. All subjects gave written informed consent in accordance with the Declaration of Helsinki. The protocol was approved by the Babes-Bolyai University IRB.

## Author Contributions

OD contributed to study design, data analysis, and manuscript writing. DD contributed to manuscript writing.

### Conflict of Interest Statement

The authors declare that the research was conducted in the absence of any commercial or financial relationships that could be construed as a potential conflict of interest.
